# Glioblastoma in the setting of prior lower grade gliomas – insights from SEER database

**DOI:** 10.18632/oncotarget.26014

**Published:** 2018-09-07

**Authors:** Ha Son Nguyen, Benjamin Best, Ninh B. Doan, Michael Gelsomino, Saman Shabani, Ahmed J. Awad, Mayank Kaushal, Martin M. Mortazavi

**Affiliations:** ^1^ Department of Neurosurgery, Medical College of Wisconsin, Milwaukee, WI, USA; ^2^ National Skull Base Center, Thousand Oaks, CA, USA; ^3^ California Institute of Neuroscience, Thousand Oaks, CA, USA; ^4^ Faculty of Medicine and Health Sciences, An-Najah National University, Nablus, Palestine; ^5^ Department of Neurosurgery, University of South Alabama, Mobile, AL, USA

**Keywords:** glioblastoma, gliosarcoma, malignant glioma, epidemiology, SEER

## Abstract

**Introduction:**

Secondary glioblastomas (GBs) constitute a small subset of all GBs and tend to arise after a lower grade glioma. Though knowledge regarding this subset has gained traction in recent years, its definition continues to evolve, complicating its clinical management. Investigation of epidemiology and survival patterns may help provide needed insights.

**Results:**

The age at GB diagnosis is significantly lower (46.22 vs 60.25 years) for group B. The distribution among type of GB (glioblastoma, giant cell glioblastoma, or gliosarcoma) was significantly different, with no diagnosis of giant cell GB in Group B. Compared to Group A, Group B exhibited a higher proportion of females, not married, smaller tumors, no GTR, and no radiation (all p < 0.05). GB-related observed survivals were comparable. Cox regression with inclusion of co-variates reveal no significant influence of GB group on observed survival. Regarding group B, mean age was 40.197 for diagnosis of initial lower grade glioma. The most common initial ICD-O-3 pathology was oligodendroglioma, NOS; astrocytoma, NOS; astrocytoma, anaplastic; and mixed glioma.

**Methods:**

The SEER-18 registry was queried for patients with GBs. Patients were further classified into two GB groups: Group A – those with GB as the only primary tumor, and Group B – those with GB as a 2^nd^ primary or subsequent tumor and with history of lower grade gliomas. Demographics and clinical factors were compared between group A and B. Appropriate statistics were employed to calculate incidences and differences among factors and GB-related survivals between the groups.

**Conclusions:**

Overall, Group B develops GBs at an earlier age, but observed survival remains similar to those with GBs as the only primary. Moreover, this subset also exhibit different proportions of the types of GBs, and well as differences in other key clinical factors (namely, gender and tumor size at presentation). Prior treatments for lower grade gliomas likely explain some of the differences noted regarding management course after diagnosis of GB.

## INTRODUCTION

Secondary glioblastomas (GBs) constitute a small subset of all GBs and tend to arise after a lower grade glioma. Though knowledge regarding this subset has gained traction in recent years, its definition continues to evolve, complicating its clinical management. Investigation of epidemiology, management, and survival patterns may help provide needed insights regarding tumor biology and treatment. Consequently, the SEER-18 registry was perused to evaluate this clinical scenario across a substantial population (~28%) of the United States [1].

## RESULTS

**Figure 1:**
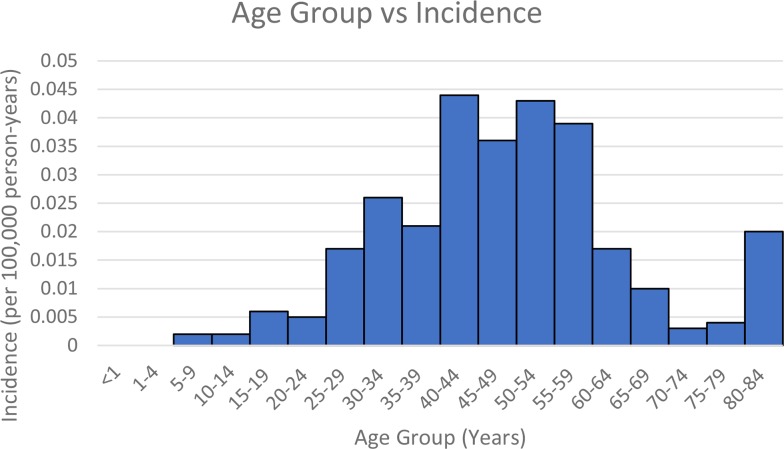
Group B – Age group vs Incidence

**Figure 2:**
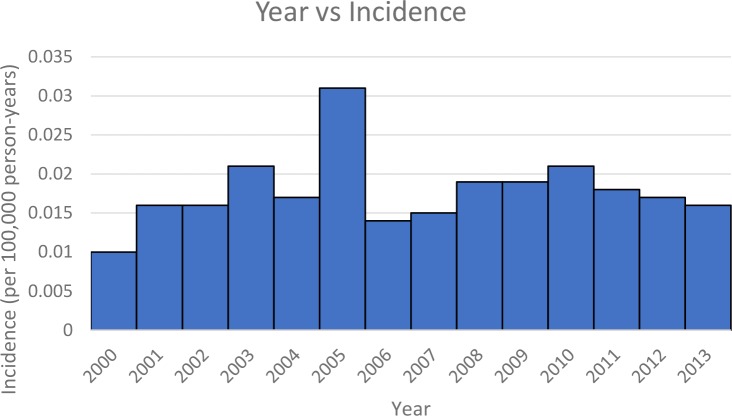
Group B: Year vs Incidence

Figure 1 shows the trend in incidence based on age for Group B. Incidence appears to have two peaks, around 40-54 and 80-84 years. Figure 2 shows the incidence by year for Group B. Comparing all values from 2001-2013 to 2000 value, 2005 value was significantly higher.

Table 1 compares the two GB groups. The age at GB diagnosis is significantly lower (46.22 vs 60.25 years) for group B. The distribution among type of GBs (glioblastoma, giant cell glioblastoma, or gliosarcoma) was significantly different, with no diagnosis of giant cell glioblastoma in Group B. Compared to Group A, Group B exhibited a higher proportion of females, not married, smaller tumors, no GTR, and no radiation (all p < 0.05). No significant difference was noted with distribution regarding presence of surgery and race. GB-related observed survivals were comparable.

**Table 1:** Comparison between GB Groups

GB Group		Group A: One primary only as GB		Group B: Subsequent GB after lower grade glioma		P - value
Age at GB diagnosis		60.25 +/- 14.567 years		46.22+/-13.043 years		<0.01
ICD-O-3	Total	42495		127		<0.01
	Glioblastoma, NOS	41210	96.98%	119	93.70%	
	Giant cell glioblastoma	406	0.96%	0	0.00%	
	Gliosarcoma	879	2.07%	8	6.30%	
Sex	Total	42495		127		0.045
	Female	17691	41.63%	64	50.39%	
	Male	24804	58.37%	63	49.61%	
Race	Total	42403		127		0.255
	Non-Caucasian	4077	9.61%	16	12.60%	
	Caucasian	38326	90.39%	111	87.40%	
Marital status	Total	41269		118		0.017
	Not married	13569	32.88%	51	43.22%	
	Married	27700	67.12%	67	56.78%	
Tumor size	Total	17605		58		<0.01
	<2.5 cm	1893	10.75%	17	29.31%	
	≥2.5cm	15712	89.25%	41	70.69%	
Surgery	Total	30156		120		0.101
	Surgery	24069	79.81%	103	85.83%	
	No surgery	6087	20.19%	17	14.17%	
GTR	Total	23724		101		0.013
	No GTR	14512	61.17%	74	73.27%	
	GTR	9212	38.83%	27	26.73%	
Radiation status	Total	40042		122		<0.01
	No	9463	23.63%	72	59.02%	
	Yes	30579	76.37%	50	40.98%	
Mean Observed Survival (months)		17.279 months		17.744 months		0.113

Table 2 lists the characteristic features of Group B when the initial lower grade glioma was diagnosed. Mean age was 40.197 years. The most common initial ICD-O-3 pathology was oligodendroglioma, NOS; astrocytoma, NOS; astrocytoma, anaplastic; and mixed glioma. For the available data, there were higher numbers of tumor size ≥ 2.5 cm compared to those < 2.5 cm. Patients predominantly underwent surgery and radiation.

**Table 2:** Characteristics of Group B

Age at diagnosis of lower grade glioma	40.197 +/- 15.2378
Pathology	
9450/3: Oligodendroglioma, NOS	39 (30.7%)
9400/3: Astrocytoma, NOS	20 (15.7%)
9401/3: Astrocytoma, anaplastic	20 (15.7%)
9382/3: Mixed glioma	12 (9.4%)
9451/3: Oligodendroglioma, anaplastic	11 (8.7%)
9391/3: Ependymoma, NOS	7 (5.5%)
9420/3: Fibrillary astrocytoma	7 (5.5%)
9392/3: Ependymoma, anaplastic	4 (3.1%)
9421/3: Pilocytic astrocytoma, malignant	3 (2.4%)
9410/3: Protoplasmic astrocytoma	2 (1.6%)
9411/3: Gemistocytic astrocytoma	1 (0.8%)
9424/3: Pleomorphic xanthoastrocytoma	1 (0.8%)
Tumor size	
<2.5 cm	9
≥2.5 cm	29
Surgery	
Surgery	82
No surgery	12
GTR	
No GTR	57
GTR	23
Radiation	
No	35
Yes	85

Table 3 delineates the Cox regression. With consideration of all other co-variates, the different GB groups did not significantly contribute to observed survival. Age, sex, race, marital status, tumor size, rate of GTR, and radiation all significantly influence observed survival.

**Table 3:** Cox regression

	HR	95.0% CI		P value
		Lower	Upper	
Age at diagnosis	1.031	1.029	1.032	<0.01
Sex	0.911	0.876	0.947	<0.01
Race	0.875	0.821	0.932	<0.01
Marital status	1.189	1.142	1.239	<0.01
Tumor size	0.891	0.837	0.949	<0.01
Surgery GTR	1.289	1.24	1.34	<0.01
Radiation	2.767	2.637	2.903	<0.01
GB Group	0.955	0.699	1.304	0.77

## DISCUSSION

The frequency of Group A drastically outnumbers Group B. Moreover, the latter exhibited a mean age roughly 14 years less than the former. This data can be paralleled to prior results regarding secondary glioblastoma (GB); our study definitions technically do not define Group B as solely secondary GB, as SEER database does not decipher this pathology as a separate entity to primary GB. However, comparison to literature on secondary GB, which has been defined by clinical history and various genetic markers, can provide some baseline. Prior studies noted that mean age for primary GB is 61-62 years, compared to 32-48 years for secondary GB [2–4], with frequency at approximately 5% of all GBs. In this SEER study, the relative ages are similar, but group B frequency is lower than reported rates for secondary GBs (0.3% vs 5%, respectively). This result could be related to the temporal advancements in the understanding of secondary GB, which began to gain traction more so during the early 2000s with the definition of genetic alterations (i.e. IDH1/2, ATRX, EGFR, PTEN, and TP53) and cDNA expression profiles. On the other hand, this SEER analysis spans 1973 to 2013, as a wide net to evaluate all GBs; therefore, GB cases recorded earlier in the collection period of the SEER data diagnosed as the only primary tumor, may in fact be a secondary GB.

Characteristics of group B align with literature regarding secondary GB. In this study, the mean latency between the diagnosis of primary lower grade glioma and GB is approximately 5 years (Table 2), like earlier studies [3]. There is also a lower ratio of males compared to females, 1.4 to 0.98 (Table 1). This trend has been reported, where male to female ratios ranged from 1.28 to 1.63 for primary GB, and 0.96 to 1.17 for secondary GB. For the most part, the listed lower grade gliomas (oligodendroglioma, NOS; astrocytoma, NOS; astrocytoma, anaplastic; mixed glioma; and ependymoma) that subsequently develop GB appear intuitive (Table 2). Moreover, Group B had a higher proportion of not married patients, which is likely related to the younger age at which the GB were diagnosed. Clinically, the data suggests that Group B presents with smaller GB tumors; though rate of surgery is equivalent to Group A, the rate of GTR is lower for Group B. It is unclear to the authors regarding the underlying explanation; since this group exhibited smaller GB tumors, the rate of GTR could be potentially higher; perhaps, the difficulty of re-do operations is a factor. The proportion utilizing radiation is lower in Group B as well, which could also be related to prior history of radiation for the lower grade glioma.

Group A has a higher proportion of giant cell glioblastoma while Group B has a higher proportion of gliosarcoma [5]. Both giant cell glioblastoma and gliosarcoma are histological variants of GB. Recent genetic studies suggest that gliosarcoma are variants of primary GB, while giant cell glioblastoma exists as an intermediate between primary and secondary GB [5]. Giant cell glioblastoma portends a more favorable survival than GB [6]. On the other hand, gliosarcoma may be a more dismal pathology, as observed survival under standard TMZ-based chemo-radiotherapy is less than GB [7]. If Group B was composed entirely of secondary GB, our results may be at odds with these studies; however, patients in Group B may have also developed primary GB (as defined by genetics) in the setting of prior lower grade glioma, which can explain the relative proportion of GB attributed gliosarcomas.

Both groups have similar GB-related observed survival (approximately 17 months). Our results from the Cox regression shows no effects on survival based on GB group (A vs B) when accommodating for other co-variates. Other co-variates (age, sex, race, marital status, tumor size, extent of tumor resection, and receipt of radiation) were significant for GB-related observed survival of the entire cohort; discussion regarding the effects of these co-variates is prevalent in the literature and will not be further elaborated here. On the other hand, it is important to note that these co-variates are significantly related with survival regardless of the diagnosis of primary and secondary GB.

Our results regarding GB group may conflict with some prior studies. In 2004, based on clinical diagnosis, Ohgaki et al [2] reported median overall survival of primary GB at 4.7 months, and secondary GB at 7.8 months; timeline was limited to the year 1999, where treatment options remained limited. Analyzing a subset of these patients based on IDH mutation (a potential marker for secondary GB), those without and with the mutation exhibited survival times at 11.3 months and 27.1 months, respectively [8]. Recently, Hamisch et al [9] only found a median survival at 11 months for secondary GB, with a cohort of 39 patients from 2004 to 2015; this result is significantly less than the 14 months reported for primary GB [10] and the 17 months observed in this study. Gessler et al [11] noted a survival around 16 months for secondary GBs for 45 patients. Overall, these differences stress the challenges associated with the diagnosis and management of secondary GB. If no genetic criteria are adopted for differentiating primary from secondary GB, no difference in outcome are seen between the two; thus, further studies on outcome should adopt more stringent diagnostic criteria than the one adopted in this study.

This study has several limitations. The SEER registry accumulates marginal data with respect to radiation, and no data regarding chemotherapy [12]. This information is necessary given the importance of multi-modal therapy in GBs. In addition, no details are provided regarding genetic / molecular profiles, such as IDH status, which can augment the results. To overcome limitations for further studies more precise data about multimodal treatment of GB and genetic/molecular profile should be included into the registry. Further studies to identify modifiable risk factors of lower grade gliomas that lead to GB should be done to maximize therapeutic opportunity for these tumors.

## MATERIALS AND METHODS

The SEER-18 registry (including Hurricane Katrina impacted Louisiana) was queried for patients with glioblastomas and its variants (ICD-O-3 histology codes 9440/3 – glioblastoma NOS, 9441/3 - giant cell glioblastoma, and 9442/3 - gliosarcoma) diagnosed from 1973 to 2013 [13–15]; this group will collectively be defined as GBs in this paper. The patients were further classified into two GB groups: Group A (reference group) – those with GB as the only primary tumor, and Group B – those with GB as a 2^nd^ primary or subsequent tumor (defined per sequence coding) and with history of lower grade gliomas (presence of prior ICD-O-3 histology codes: 9380 glioma, 9381 gliomatosis, 9382 mixed glioma, 9383 subependymoma, 9384 SEGA, 9391-9394 ependymoma, 9400-9401 astrocytoma, 9410 protoplasmic astrocytoma, 9411 gemistocytic astrocytoma, 9412 desmoplastic infantile astrocytoma, 9420 fibrilillary astrocytoma, 9421 pilocytic astrocytoma, 9424 pleomorphic xanthoastrocytoma, 9444 chondroid glioma, and/or 9450-9451 oligodendroglioma]).

Only cases with microscopically confirmed / actively followed/ known age / within research database were considered; death certificate only/ autopsy only / alive with no survival time were excluded [13–15]. Age, gender, race, marital status, sequence of diagnosis relative to other primary tumors, occurrence and extent of surgery, tumor size, receipt of radiation, and follow-up data were acquired.

With the SEER^*^Stat software, the following calculations were conducted for these two sequence groups A and B [13–15]: 1) incidence rates (per 100,000 person-years) by age groups and 2) incidence rates by year from 2000-2013.

Demographics and clinical factors were compared between group A and B. Age was treated as a continuous variable. Gender was dichotomized to males and females. Marital status was dichotomized to single (coded as “single, never married”, “separated”, “divorced”, “widowed”, or “unmarried or domestic partner”) and married (coded as “married, including common law). Race was dichotomized to Caucasian (coded as “white”) or Non-Caucasian (coded as “black”, “American Indian / Alaska Native”, or “Asian or Pacific Islander”). Tumor was dichotomized with a threshold of 2.5 cm. Any case with an unknown value for a specific variable was excluded from the analysis of that specific variable.

Occurrence of surgery was defined as such [13–15]: “No surgery” – those coded as “no surgery (00)” OR “Surgery” – those coded as local tumor destruction NOS (10), biopsy (20), surgery NOS (90), partial resection NOS (40), and subtotal resection (21), gross total resection (55), or radical, total, gross total resection (30). Of those who underwent surgery, the extent of primary surgery was defined as follows, similar to previously described [13–15]: “No GTR” – those coded as “local tumor destruction NOS (10), biopsy (20), partial resection NOS (40), and subtotal resection (21)” OR “GTR” – those coded as gross total resection (55) or radical, total, gross total resection (30). “Surgery status unknown” – those coded as surgery, unknown (99) – and surgery NOS (90) was not included in the extent of resection analysis.

Receipt of radiation was defined as follows [13–15]: “No radiation” – those coded as none (0) and patient or patient’s guardian refused radiation therapy (7) OR “Radiation” – those coded as beam radiation (1), radioactive implants (2), radioisotopes (3), combination of 1 with 2 or 3 (4), and radiation NOS (5); unknown status of radiation was not included in the relevant analysis.

IBM SPSS 22 was utilized for statistical analysis to evaluate the difference between the two groups A and B. Pearson’s chi-squared (χ^2^) test or Fisher’s exact test was employed to evaluate categorical variables and student t test was employed for continuous variables. GB-related observed survival was determined using the Kaplan–Meier method and compared via log-rank test. Multivariate analysis was conducted via Cox proportional hazards model to evaluate the influence of GB groups relative to other co-variates on observed survival of all GBs. All p values reported represent two-sided statistical tests. A p < 0.05 were considered statistically significant. Categorical data were conveyed via frequency counts with percentages. Continuous data were conveyed via mean values with standard deviations.

## CONCLUSIONS

Gliomas have a spectrum of invasiveness. Knowledge regarding patients with lower grade gliomas with subsequent GB is crucial to understand tumor pathophysiology. Based on our analysis from the SEER registry, this subset of patients develops GB at an earlier age, but observed survival remains similar to those with de novo GB. Moreover, this subset also exhibits different proportions of the GB variants, as well as differences in other key clinical factors (namely, gender and tumor size at presentation). Prior treatments for lower grade gliomas likely explain some of the differences noted regarding management course after diagnosis of GB. Important co-variates (such as age, sex, race, marital status, tumor size, extent of tumor resection, and receipt of radiation) are significantly related with observed survival regardless of the diagnosis of primary and secondary GB. Caution is stressed, as the SEER registry does have limitations as noted; for subsequent data collection and further studies, more precise data about multimodal treatment of GB and genetic/molecular profile should be included into the registry.
